# Supplementary material for: MetaRibo-Seq measures translation in microbiomes
Source: Nat Commun. 2020 Jun 29;11:3268. doi: 10.1038/s41467-020-17081-z (PMC7324362; doi:10.1038/s41467-020-17081-z)
Supplement: Supplementary file 10 — Supplementary Data 7 [file 41467_2020_17081_MOESM10_ESM.zip › File2/Confidence_High_Taxonomy/182698_out.krona.html]

Javascript must be enabled to view this page.

members
magnitude
magnitudeUnassigned
count
unassigned
taxon
rank

182698\_out

9

9
superkingdom
2

9
976
phylum

9
class
200643

order
171549
9

9
family
815

9
816
genus

4
species
28116

SRS024663\_contig\_number\_14100SRS043001\_contig\_number\_2230SRS098571\_contig\_number\_29042SRS149879\_contig\_number\_28187

2
species

SRS016267\_contig\_number\_6538SRS1041130\_contig\_number\_5717
2292951

3

SRS043667\_contig\_number\_6539SRS075341\_contig\_number\_21344SRS075878\_contig\_number\_contig-100\_221.138517
371601
species
